# Evaluation of a Novel Semiquantitative Cryptococcal Antigen Lateral Flow Assay in Patients with Advanced HIV Disease

**DOI:** 10.1128/JCM.00441-20

**Published:** 2020-08-24

**Authors:** Joseph N. Jarvis, Mark W. Tenforde, Kwana Lechiile, Thandi Milton, Amber Boose, Tshepo B. Leeme, Leabaneng Tawe, Charles Muthoga, Ivy Rukasha, Fredah Mulenga, Ikanyeng Rulaganyang, Mooketsi Molefi, Síle F. Molloy, Julia Ngidi, Thomas S. Harrison, Nelesh P. Govender, Madisa Mine

**Affiliations:** a Botswana Harvard AIDS Institute Partnership, Gaborone, Botswana; b Department of Clinical Research, Faculty of Infectious and Tropical Diseases, London School of Hygiene and Tropical Medicine, London, UK; c Botswana University of Pennsylvania Partnership, Gaborone, Botswana; d Division of Allergy and Infectious Diseases, Department of Medicine, University of Washington School of Medicine, Seattle, Washington, USA; e Department of Epidemiology, University of Washington School of Public Health, Seattle, Washington, USA; f National Institute for Communicable Diseases, National Health Laboratory Service, Johannesburg, South Africa; g Botswana National Health Laboratory, Gaborone, Botswana; h Faculty of Medicine, University of Botswana, Gaborone, Botswana; i Centre for Global Health, Institute of Infection and Immunity, St George’s University of London, London, UK; j School of Pathology, Faculty of Health Sciences, University of the Witwatersrand, Johannesburg, South Africa; University of Utah

**Keywords:** Cryptococcal meningitis, HIV, cryptococcal antigen, diagnostic accuracy, lateral flow assay, validation study

## Abstract

Higher cryptococcal antigen (CrAg) titers are strongly associated with mortality risk in individuals with HIV-associated cryptococcal disease. Rapid tests to quantify CrAg levels may provide important prognostic information and enable treatment stratification. We performed a laboratory-based validation of the IMMY semiquantitative cryptococcal antigen (CrAgSQ) lateral flow assay (LFA) against the current gold standard CrAg tests. We assessed the diagnostic accuracy of the CrAgSQ in HIV-positive individuals undergoing CrAg screening, determined the relationship between CrAgSQ scores and dilutional CrAg titers, assessed interrater reliability, and determined the clinical correlates of CrAgSQ scores. A total of 872 plasma samples were tested using both the CrAgSQ LFA and the conventional IMMY CrAg LFA, of which 692 were sequential samples from HIV-positive individuals undergoing CrAg screening and an additional 180 were known CrAg-positive plasma samples archived from prior studies. Interrater agreement in CrAgSQ reading was excellent (98.17% agreement, Cohen’s kappa 0.962, *P* < 0.001). Using the IMMY CrAg LFA as a reference standard, CrAgSQ was 93.0% sensitive (95% confidence interval [CI] 80.9% to 98.5%) and 93.8% specific (95% CI, 91.7% to 95.6%). After reclassification of discordant results using CrAg enzyme immunoassay testing, the sensitivity was 98.1% (95% CI, 90.1% to 100%) and specificity 95.8% (95% CI, 93.9% to 97.2%). The median CrAg titers for semiquantitative score categories (1+ to 4+) were 1:10 (interquartile range [IQR], 1:5 to 1:20) in the CrAgSQ 1+ category, 1:40 (IQR, 1:20 to 1:80) in the CrAgSQ 2+ category, 1:640 (IQR, 1:160 to 1:2,560) in the CrAgSQ 3+ category, and 1:5,120 (IQR, 1:2,560 to 1:30,720) in the CrAgSQ 4+ category. Increasing CrAgSQ scores were strongly associated with 10-week mortality. The IMMY CrAgSQ test had high sensitivity and specificity compared to the results for the IMMY CrAg LFA and provided CrAg scores that were associated with both conventional CrAg titers and clinical outcomes.

## Abstract (precis)

Higher cryptococcal antigen (CrAg) titers are strongly associated with mortality risk in individuals with HIV-associated cryptococcal disease. Rapid tests to quantify CrAg levels may provide important prognostic information and enable treatment stratification. We performed a laboratory-based validation of the IMMY semiquantitative cryptococcal antigen (CrAgSQ) lateral flow assay (LFA) against the current gold standard CrAg tests. We assessed the diagnostic accuracy of the CrAgSQ in HIV-positive individuals undergoing CrAg screening, determined the relationship between CrAgSQ scores and dilutional CrAg titers, assessed interrater reliability, and determined the clinical correlates of CrAgSQ scores.

## INTRODUCTION

Cryptococcal meningitis is a major cause of morbidity and mortality among people living with HIV, resulting in an estimated 15% of HIV-related deaths worldwide (1). The incidence of HIV-associated cryptococcal meningitis has remained high in many low- and middle-income settings despite improved population-level access to antiretroviral therapy (ART) (1–3). Mortality rates from cryptococcal meningitis with currently available treatments also remain unacceptably high, ranging from 25% to 45% at 10 weeks (4–7). There is an urgent need to improve both prevention and treatment strategies (8, 9).

Detection of the cryptococcal capsular polysaccharide antigen glucuronoxylomannan (GXM), commonly known as cryptococcal antigen (CrAg), in body fluids including cerebrospinal fluid (CSF) and blood (whole blood, plasma, or serum) is the cornerstone of diagnosis. Highly sensitive and specific CrAg lateral flow assays (LFAs) (10, 11), appropriate for use in laboratories with limited facilities or at the point of care (12), have markedly facilitated rapid clinical diagnosis of cryptococcal meningitis and also enabled the implementation of CrAg screening programs aimed at detecting and treating early asymptomatic infection in HIV-positive individuals with low CD4 T-cell counts (13–16). Beyond a qualitative (positive/negative) result, higher CrAg titers have been shown to be strongly associated with increased fungal burden and mortality risk in patients with cryptococcal meningitis (17) and with the presence of central nervous system (CNS) infection and mortality risk among asymptomatic CrAg-positive individuals identified through blood/plasma CrAg screening programs (18–20). Determining CrAg titers may therefore provide important prognostic information, enabling stratification of treatment in individuals with cryptococcal meningitis and identification of CrAg-positive individuals detected through CrAg screening programs who require lumbar puncture (LP) to rule out CNS infection or may benefit from more intensive antifungal therapy.

**FIG 1:**
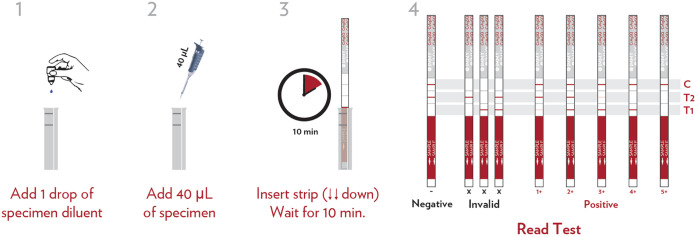
The IMMY semiquantitative CrAgSQ lateral flow assay (IMMY, Norman, OK, USA). Samples are diluted 1:1 with specimen diluent prior to testing (as is also the case with the conventional lateral flow assay). Scores indicating increasing cryptococcal antigen titers are derived from line intensity patterns as follows: T1 < T2 = 1+; T1 = T2 = 2+; T1 > T2 = 3+; only T1 = 4+; only C = 5+; and only T2 and C = negative. A score of 1+ indicates “low positive,” and a score of 5+ indicates “very high positive.”

The currently used commercial CrAg assays provide a qualitative dichotomous positive/negative result, necessitating the testing of serial dilutions of the primary specimen to obtain a quantitative titer result. This serial testing involves significant additional costs, is time intensive, and requires laboratory operator expertise that limits performance in clinical practice, and therefore, it is rarely performed in low-resource settings. To overcome these limitations, IMMY (Norman, OK, USA) has developed the CrAgSQ LFA, a new immunochromatographic test system for the semiquantitative (SQ) detection of the capsular polysaccharide antigens of the Cryptococcus neoformans*/gattii* species complex in serum, plasma, whole blood, and CSF (Fig. 1). We performed a comprehensive laboratory-based validation study to evaluate the performance of the CrAgSQ assay against the current gold standard CrAg detection tests, assessing the diagnostic accuracy of the CrAgSQ assay in HIV-positive individuals undergoing CrAg screening, determining the relationship of CrAgSQ band cutoffs with conventionally derived CrAg titers, assessing interrater reliability to inform interpretability of the CrAgSQ assay, and determining clinical correlates of CrAgSQ results.

## MATERIALS AND METHODS

### Study population and procedures.

**FIG 2:**
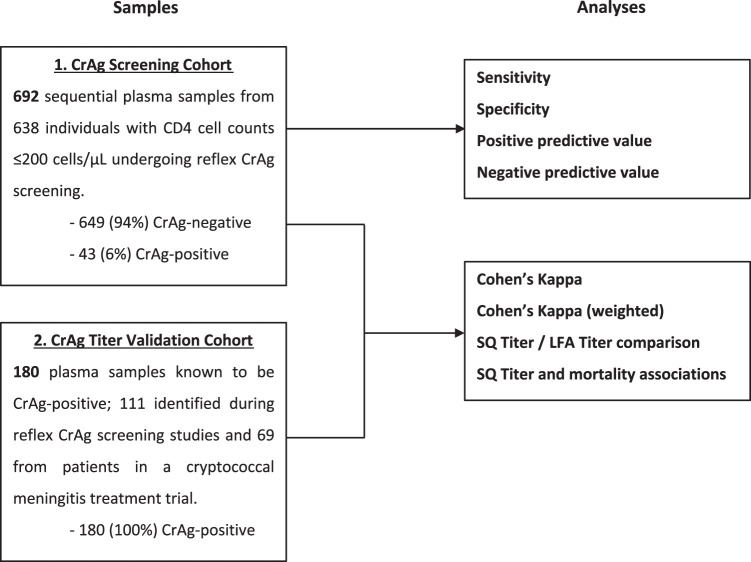
Schema of plasma samples and patient populations used in the diagnostic validation study.

The study was performed in two parts (Fig. 2). In the first part, the qualitative (positive/negative) performance of the CrAgSQ assay was evaluated against the first-generation qualitative IMMY CrAg LFA in samples from a consecutively recruited cohort of HIV-positive individuals with CD4 cell counts of ≤200 cells/μl undergoing reflex CrAg screening at the Botswana-Harvard HIV Reference Laboratory (BHHRL) in Gaborone, Botswana, between January and August 2018 (cohort 1). BHHRL performs almost all CD4 testing for 27 public ART clinics and a national referral hospital in greater Gaborone. Residual EDTA whole blood sent to the BHHRL for routine CD4 testing found to have a CD4 cell count of ≤200 cells/μl underwent CrAg screening using the IMMY CrAg LFA as part of a reflex CrAg screening study. The IMMY CrAg LFA is an immunochromatographic CrAg test that provides a qualitative test result within 15 min. The assay has been extensively validated on whole-blood, serum, plasma, and CSF samples and is highly accurate compared to other commercial assay types, such as available enzyme immunoassays (EIAs) (10, 11). Plasma was separated and stored in −80°C freezers at the Botswana National Health Laboratory for subsequent CrAgSQ testing and IMMY CrAg LFA titer determination on completion of study recruitment. The sensitivity and specificity of the CrAgSQ were determined against the IMMY CrAg LFA as a reference standard, with all positive CrAgSQ scores (1+, 2+, 3+, 4+, or 5+) considered positive results.

The second part of the study evaluated the relationship between CrAgSQ semiquantitative results (CrAgSQ score) and (i) IMMY CrAg LFA titers and (ii) clinical outcome (CNS disease in the CrAg-screened population and 10-week mortality in all cases). These assessments were performed using the plasma samples from cohort 1 described above plus a collection of stored frozen plasma samples that had previously tested CrAg positive using the IMMY CrAg LFA (cohort 2). Cohort 2 consisted of all plasma samples with positive CrAg tests from a 2015–2016 CrAg screening cohort study performed at BHHRL (20) and from a phase II randomized controlled trial (RCT) evaluating cryptococcal meningitis therapies in Gaborone, Botswana (21).

All CrAgSQ assays were performed by trained laboratory technicians according to the manufacturer’s instructions and read by two independent laboratory technicians blinded to previous CrAg test results, as well as the other technician’s read. Results were recorded as negative or 1+, 2+, 3+, 4+, or 5+ (Fig. 1). The CrAgSQ test turnaround time is approximately 15 min, similar to the conventional IMMY LFA, and relies on a visual reading of line color intensity; thus, it is subject to a degree of operator dependency. Discordant reads between technicians were arbitrated by a third investigator, and a photographic record of all test strips was maintained (see the supplemental material). Samples with discrepant qualitative results from the IMMY LFA and CrAgSQ assays, i.e., one positive and the other negative, were retested at an independent accredited laboratory using a commercial enzyme immunoassay (EIA) (IMMY, Norman, OK, USA) by a scientist blinded to previous results.

Patients identified as CrAg positive during the CrAg screening studies were treated according to an algorithm based on World Health Organization (WHO) guidelines (9,11), recommending high-dose fluconazole (1,200 mg/day) for asymptomatic individuals, LP to rule out CNS infection, and referral for amphotericin B-based treatment as an inpatient if CSF was CrAg positive (Fig. S1 in the supplemental material). In the 2015–2016 screening study, management decisions were at the discretion of the patients’ health care providers and the research team had no direct patient contact; thus, they were unable to directly assess adherence to treatment guidelines. In the 2018 screening study, the research team actively managed CrAg-positive patients. Patient follow-up data were collected to 6 months. The phase II RCT has been described in detail elsewhere (21). Participants were treated with either amphotericin B deoxycholate or liposomal amphotericin B, both given with fluconazole, as inpatients and actively followed up to 10 weeks.

The research was approved by Institutional Review Boards at the University of Botswana, the Botswana Ministry of Health and Wellness, and the University of Pennsylvania. As the two CrAg screening studies were limited to implementation of a laboratory-based, WHO-endorsed screening intervention and collection of routine clinical and outcome data, a waiver of informed patient consent was granted. All patients in the phase II treatment trial provided written informed consent providing permission for sample storage and testing.

### Validation study analysis.

**(i) Sensitivity and specificity.** Using the cohort 1 samples, we calculated the sensitivity, specificity, and positive and negative predictive values of the CrAgSQ LFA for qualitative (positive/negative) CrAg detection in plasma using the first-generation IMMY LFA as the reference standard. In order to better classify potential false-positive and false-negative results, an additional analysis was performed in which the IMMY EIA result was used as a tiebreaker for samples with discrepant test results between the CrAgSQ assay and IMMY CrAg LFA, and the reference test result reclassified accordingly. Sensitivity, specificity, and positive and negative predictive values were recalculated against this tiebreaker-adjusted composite reference standard.

**(ii) Interrater reliability.** We assessed interrater reliability for the CrAgSQ assay using all samples from cohort 1 and cohort 2. Percent agreement between the two reading technicians was determined using an unadjusted Cohen’s kappa statistic. As the CrAgSQ test has ordered categorical values, we calculated a second weighted kappa with 75% weight given to values that were within 1 category between the two reviewers, (e.g., readings of 3+ and 4+), 50% weight given to values within 2 categories, 25% weight within 3 categories, and 0% weighting for a discrepancy of four categories (22).

**(iii) Association between CrAgSQ quantification and CrAg titers.** To evaluate the associations between semiquantitative CrAgSQ LFA results and CrAg titer values, median CrAg titers were calculated in each CrAgSQ score category using all samples from cohorts 1 and 2, and the results displayed graphically.

**(iv) Relationship between CrAgSQ results and clinical outcomes.** The proportion of CrAg-screened individuals with confirmed CNS infection (cryptococcal meningitis) at baseline in cohorts 1 and 2 (excluding those in the phase II treatment trial) and the overall proportion of individuals who died by 10 weeks were calculated according to plasma CrAgSQ score category, and the association between plasma CrAgSQ results and 10-week mortality examined using a Cox proportional hazards model. A sensitivity analysis was performed in which all individuals lost to follow-up were assumed to have died.

All analyses were performed using STATA version 14 (Stata Corporation, College station, TX). *P* values of <0.05 were considered significant.

## RESULTS

A total of 872 plasma samples were tested using both the IMMY CrAgSQ and the IMMY CrAg LFA; 692 were from cohort 1 and 180 from cohort 2 (Fig. 2). Baseline characteristics of the study participants are shown in Table 1. Interrater agreement in CrAgSQ reading was excellent, with 98.2% agreement, Cohen’s kappa of 0.96, and *P* < 0.001 (Tables 2 and 3).

**TABLE 1:** Baseline characteristics of study participants

Variable^*a*^	Value [median (IQR) or % (no. of individuals)]
Cohort 1^*b*^	*n* = 638
Age (yrs)	40 (33–46)
Sex (% male)	57 (365)
CD4 count (cells/μl)	91 (53–150)
Cryptococcal antigenemia (% positive)^*c*^	5.8 (37)
Testing location (% outpatients)	88 (563)
ART status (% on ART)^*d*^	71 (451)
Prior cryptococcal meningitis (%)	2 (13)

Cohort 2^*e*^	*n* = 180
Age (yrs)	39 (34–44)
Sex (% male)	59 (104)
CD4 count (cells/μl)	41 (16–85)
Cryptococcal antigenemia (% positive)^*c*^	100 (180)
Testing location (% outpatients)	49 (88)
ART status (% on ART)^*f*^	46 (82)
Prior cryptococcal meningitis (%)	12 (21)

a IQR, interquartile range; CrAg, cryptococcal antigen; ART, antiretroviral therapy.

b Sequential cohort of individuals with CD4 cell counts of ≤200 cells/μl whose test samples were obtained during a reflex CrAg screening program.

c Cryptococcal antigen positive using the IMMY CrAg lateral flow assay (IMMY, Norman, OK, USA).

d Among the participants, 451/638 (71%) were on ART, 32/638 (5%) defaulted ART, and 155/638 (24%) were ART naive. Viral loads were available for 448 of those on ART; 168 (38%) had a detectable viral load.

e CrAg titer validation cohort that consisted of 111 known-CrAg-positive plasma samples from reflex cryptococcal antigen screening studies and 69 plasma samples from patients with cryptococcal meningitis enrolled in a clinical trial (21).

f Among the participants, 82/180 (46%) were on ART, 13/180 (7%) defaulted ART, and 85/180 (47%) were ART naive. Viral loads were available for 55 of those on ART; 16 (29%) had a detectable viral load.

**TABLE 2:** Diagnostic performance of the IMMY CrAgSQ LFA for interrater agreement

Score from rater A	No. of tests with score from rater B					Total
	0	1+	2+	3+	4+	
0	610	0	0	0	0	610
1+	4	87	0	0	0	91
2+	0	3	15	0	0	18
3+	0	0	4	101	5	110
4+	0	0	0	0	43	43

Total	614	90	19	101	48	872

**TABLE 3:** Diagnostic performance of the IMMY CrAgSQ LFA for interrater reliability

Interrater reliability	% agreement		Cohen’s kappa	SE	*P* value
	Expected	Observed			
Unweighted	52.11	98.17	0.962	0.022	<0.0001
Weighted^*a*^	72.07	99.54	0.983	0.028	<0.0001

a To account for the ordered categorical data and assess the degree of disagreement, disagreements were weighted in a linear way: with five categories, cases in adjacent categories were weighted by a factor of 0.75, those with a distance of two categories by a factor of 0.5, those with a distance of three categories by a factor of 0.25, and those with a distance of four categories by a factor of 0.

Of the 692 samples tested in the sequentially enrolled cohort 1, 43 (6.2%) were positive for CrAg using the IMMY CrAg LFA. Compared to the IMMY CrAg LFA as a reference standard, CrAgSQ (used as a qualitative test) was 93.0% sensitive (95% confidence interval [CI], 80.9% to 98.5%) and 93.8% specific (95% CI, 91.7% to 95.6%) (Table 4). Forty of the 649 (6.2%) IMMY CrAg LFA-negative samples were positive on CrAgSQ testing, all at the lowest 1+ score, and classified as false positive; 3 of the IMMY CrAg LFA-positive samples (all with the lowest titer of 1:2) were negative on CrAgSQ testing and classified as false negative.

**TABLE 4:** Diagnostic performance of the IMMY CrAgSQ LFA versus the conventional IMMY CrAg LFA for sensitivity, specificity, and positive and negative predictive values

CrAgSQ LFA result	No. of tests with indicated result in CrAg LFA^*a*^		Total	Value [% (95% CI)] for^*b*^:			
	Positive	Negative		Sensitivity	Specificity	PPV	NPV
Positive^*c*^	40	40	80	93.0 (80.9–98.5)	93.8 (91.7–95.6)	50.0 (38.6–61.4)	99.5 (98.6–99.9)
Negative	3	609	612				

Total	43	649	692				

a The conventional IMMY qualitative lateral flow assay (LFA) was used as the reference test against the IMMY semiquantitative LFA for CrAg testing.

b PPV, positive predictive value; NPV, negative predictive value.

c All CrAgSQ results of 1+ and above were considered positive.

**FIG 3:**
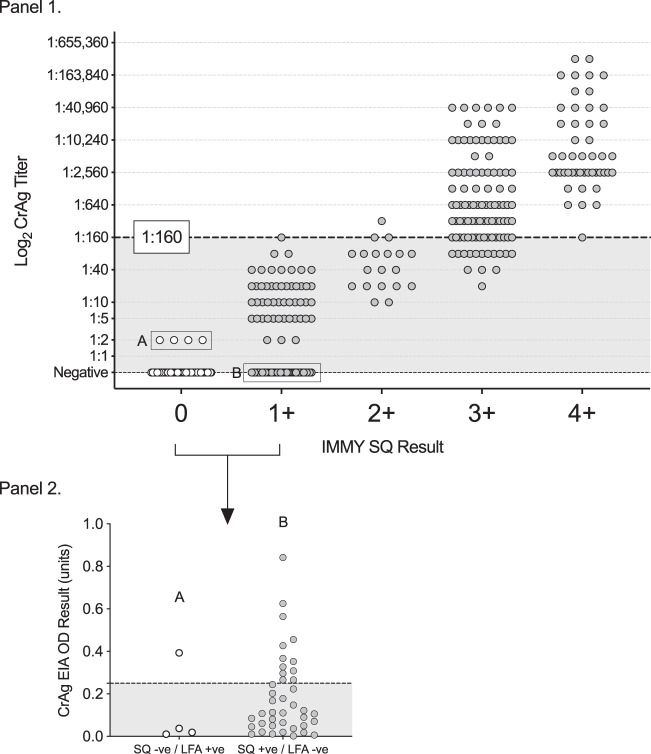
Relationship between CrAgSQ scores and cryptococcal antigen titers derived from serial dilutional testing with the IMMY lateral flow assay (panel 1). Samples with discordant CrAgSQ and IMMY lateral flow assay positive/negative results were retested using the IMMY cryptococcal antigen enzyme immunoassay (EIA) (panel 2). Box A indicates samples that were positive on IMMY lateral flow assay testing and negative on CrAgSQ testing. Three of these four samples were negative on EIA testing at the optical density cutoff of 0.265. Box B indicates the samples that were positive on CrAgSQ testing and negative on IMMY lateral flow assay testing. Thirteen of the forty samples were positive on EIA testing at the optical density cutoff of 0.265. −ve, negative; +ve, positive.

On EIA testing, 13 of the 40 CrAgSQ false-positive samples were found to be CrAg positive, with EIA readings above the optical density cutoff of 0.265 as specified by the manufacturer, and thus, were reclassified as true positives. Two of the 3 CrAgSQ false negatives were negative on EIA testing and, thus, reclassified as true negatives (Fig. 3). Following this adjustment to the reference standard, the sensitivity of the CrAgSQ was 98.1% (95% CI, 90.1% to 100%) and the specificity was 95.8% (95% CI, 93.9% to 97.2%) (Table 5); 27 (4.2%) of the IMMY CrAg LFA- and EIA-negative samples were CrAgSQ positive, all at the lowest 1+ score, and classified as false positive, and 1 of the IMMY CrAg LFA- and EIA-positive samples (at the lowest titer of 1:2) was negative on CrAgSQ testing and classified as false negative. Detailed clinical information for these unreconciled discordant results (27 false positives and 1 false negative) is shown in Table S1.

**TABLE 5:** Diagnostic performance of the IMMY CrAgSQ LFA versus the conventional IMMY CrAg LFA for sensitivity, specificity, and positive and negative predictive values after reconciliation of discordant test results using EIA

CrAgSQ result	No. of tests with indicated tiebreaker-adjusted CrAg result^*a*^		Total	Value [% (95% CI)] for^*b*^:			
	Positive	Negative		Sensitivity	Specificity	PPV	NPV
Positive^*c*^	53	27	80	98.1 (90.1–100)	95.8 (93.9–97.2)	66.3 (54.8–76.4)	99.8 (99.1–100)
Negative	1	611	612				

Total	54	638	692				

a The reference standard was a composite cryptococcal antigen (CrAg) result derived from the conventional IMMY qualitative lateral flow assay (LFA) with discrepant conventional CrAG LFA/CrAgSQ (semiquantitative) LFA results reconciled using the IMMY enzyme immunoassay (EIA) as the tiebreaker test. See Fig. 3 for details.

b PPV, positive predictive value; NPV, negative predictive value.

c All CrAgSQ results of 1+ and above were considered positive.

Combining all 223 CrAg-positive plasma samples from cohort 1 (*n* = 43) and cohort 2 (*n* = 180), the median CrAg titers were 1:10 (interquartile range [IQR], 1:5 to 1:20) in the CrAgSQ 1+ category, 1:40 (IQR, 1:20 to 1:80) in the CrAgSQ 2+ category, 1:640 (IQR, 1:160 to 1:2,560) in the CrAgSQ 3+ category, and 1:5,120 (IQR, 1:2,560 to 1:30,720) in the CrAgSQ 4+ category (Fig. 3).

**FIG 4:**
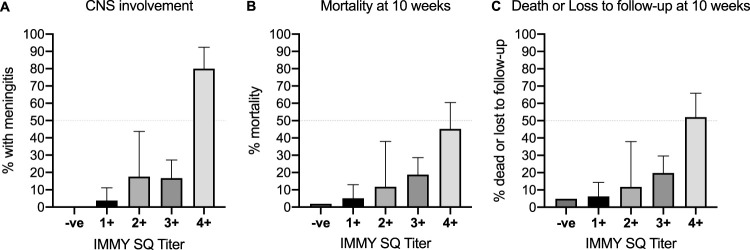
Associations between CrAgSQ score and baseline CNS disease (defined as a CrAg-positive cerebrospinal fluid sample) in the 189 CrAg-positive patients identified through reflex cryptococcal antigen screening (A), 10-week mortality in all participants (B), and 10-week mortality and loss to follow-up (C). Note that only 32% of CrAg-positive individuals underwent baseline CSF examination, and thus, these figures represent minimum estimates of baseline CNS disease.

Among the 189 CrAgSQ-positive patients included in the two CrAg screening studies (excluding the cryptococcal meningitis patients enrolled in the treatment trial), the prevalence of CNS involvement at baseline was strongly associated with the CrAgSQ score. Cryptococcal meningitis was confirmed at baseline in 3.8% (3/80) in the CrAgSQ 1+ category, 17.7% (3/17) in the 2+ category, 16.7% (12/72) in the 3+ category, and 80% (16/20) in the 4+ category, with a *P* value of <0.001 for the trend (Fig. 4); it is important to note that these are minimum estimates, as LP was only performed in approximately one-third of patients (primarily because patients declined the investigation) and possibly performed more frequently in those with symptoms of CNS disease. The CrAgSQ score was also strongly associated with mortality. Overall in the combined cohorts 1 and 2, 10-week mortality was 2.0% (11/554) in CrAgSQ-negative individuals, 5.1% (4/78) in those with CrAgSQ 1+ scores, 11.8% (2/17) in those with CrAgSQ 2+ scores, 18.8% (16/85) in those with CrAgSQ 3+ scores, and 45.2% (19/42) in those with CrAg 4+ scores (Table 6), with a *P* value of <0.0001. Restricting analysis to participants in the two CrAg screening studies (excluding the cryptococcal meningitis patients enrolled in the treatment trial), 10-week mortality was 2.0% (11/554) in CrAgSQ-negative individuals, 2.9% (2/68) in those with CrAgSQ 1+ scores, 13.3% (2/15) in those with CrAgSQ 2+ scores, 16.1% (9/56) in those with CrAgSQ 3+ scores, and 53.3% (8/15) in those with CrAg 4+ scores (Table 6), with a *P* value of <0.001. The findings in sensitivity analyses where those lost to follow-up were assumed to have died were unchanged (Table 6).

**TABLE 6:** Associations between IMMY CrAgSQ LFA titers and mortality

Group analyzed	CrAgSQ score	% mortality (no. of deaths/total no. of participants)	Hazard ratio^*a*^	95% CI	*P* value
CrAg screening and RCT participants, including^*b*^:					
Confirmed deaths^*c*^	0	2.0 (11/554)	Base		<0.0001
	1+	5.1 (4/78)	2.63	0.84–8.26	
	2+	11.8 (2/17)	6.26	1.39–28.25	
	3+	18.8 (16/85)	10.18	4.73–21.95	
	4+	45.2 (19/42)	28.85	13.70–60.75	

Those dead or lost to follow-up^*d*^	0	4.9 (28/571)	Base		<0.0001
	1+	6.3 (5/79)	1.29	0.50–3.34	
	2+	11.8 (2/17)	2.45	0.59–10.31	
	3+	19.8 (17/86)	4.12	2.30–7.67	
	4+	52.1 (25/48)	14.1	8.19–24.17	

CrAG screening participants only, including^*e*^:					
Confirmed deaths^*b*^	0	2.0 (11/554)	Base		<0.0001
	1+	2.9 (2/68)	1.75	0.50–6.13	
	2+	13.3 (2/15)	5.70	1.29–25.27	
	3+	16.1 (9/56)	6.85	3.00–15.62	
	4+	53.3 (8/15)	31.80	13.01–77.31	

Those dead or lost to follow-up^*c*^	0	4.9 (28/571)	Base		<0.0001
	1+	4.4 (3/69)	0.97	0.35–2.77	
	2+	13.3 (2/15)	2.38	0.57–9.94	
	3+	17.5 (10/57)	3.11	1.57–6.20	
	4+	65.0 (13/20)	18.33	9.54–35.22	

a Derived from Cox proportional hazards model.

b Analysis included participants in the two CrAg screening studies and the cryptococcal meningitis patients enrolled in the phase II treatment trial (21). RCT, randomized controlled trial.

c Mortality at 10 weeks with loss to follow-ups censored.

d Dead or lost to follow-up at 10 weeks: 25 patients (3%) were lost to follow-up prior to 10 weeks.

e Analysis was restricted to participants in the two CrAg screening studies and excluded the cryptococcal meningitis patients enrolled in the phase II randomized controlled treatment trial (21).

## DISCUSSION

The novel CrAgSQ semiquantitative CrAg LFA had high sensitivity and specificity when compared to the current gold standard CrAg LFA. Interrater agreement in reading the semiquantitative results was excellent, and the test provided rapid and reliable estimation of CrAg titers. Increasing CrAgSQ scores were strongly associated with the presence of CNS involvement in CrAg-positive individuals identified through CrAg screening programs and with acute mortality.

The role of CrAg titers in guiding clinical management of HIV-positive patients with asymptomatic cryptococcal antigenemia identified through CrAg screening programs and in those with overt clinical cryptococcal meningitis has yet to be defined. Accumulating clinical data suggest that quantification of CrAg levels using tests like the CrAgSQ could enable stratification of patients into differentiated diagnostic and treatment pathways. Recent data from CrAg screening programs in Africa have shown that asymptomatic CrAg-positive individuals with CD4 cell counts below 200 cells/μl have mortality rates 2- to 3-fold higher than their CrAg-negative counterparts with similar CD4 counts (20, 23, 24), despite treatment with high-dose oral fluconazole therapy as recommended in WHO guidelines (16). This is likely to be due in part to the presence of CNS disease, detectable by LP and CSF evaluation, in approximately one-third of asymptomatic CrAg-positive patients with advanced HIV (18), for whom fluconazole monotherapy is likely to be insufficient to effectively clear infection (25, 26). However, even CrAg-positive individuals without CNS involvement at baseline have been shown to progress to cryptococcal meningitis and death despite high-dose fluconazole therapy (27), suggesting that a proportion of CrAg-positive patients without overt CNS disease may benefit from the intensified antifungal regimens recommended for the treatment of cryptococcal meningitis (16, 28). Conversely, it is well established that a sizeable proportion of asymptomatic CrAg-positive individuals identified through screening programs (approximately 50%) can clear their cryptococcal antigenemia with effective ART-induced immune reconstitution alone (13).

Identifying which CrAg-positive individuals require investigation for CNS disease and/or more intensive antifungal therapy regimens and which can be managed effectively with oral fluconazole alone is therefore a critical question for CrAg screening programs (16). Elevated CrAg titers of >1:160 have been shown to be highly predictive of prevalent CNS disease at the time of CrAg screening (with a sensitivity of 88% and specificity of 82%) in a study from South Africa (18), with similar findings reported in Ethiopia (29). Elevated CrAg titers of ≥1:80 or ≥1:160 have also been shown to be strongly associated with increased risk of mortality in CrAg-positive populations (19, 20). Our findings that higher CrAgSQ antigen quantification scores are strongly associated with both CNS involvement at baseline in CrAg-positive individuals and higher mortality at 10 weeks add to the already compelling evidence that CrAg titer data could be used to risk stratify CrAg-positive individuals and guide treatment. Although further data are required to inform definitive clinical guidelines, a preliminary suggestion based on our data could be that individuals with a CrAgSQ 1+ score, in whom the risk of baseline CNS involvement and acute mortality is very low, could be managed according to current guidelines with high-dose fluconazole and ART alone, without the need for LP. Those with CrAgSQ scores of 2+ to 3+ could undergo more intensive clinical evaluation and/or receive more intensive antifungal therapy, for example, a combination of fluconazole and flucytosine (4), while those with CrAgSQ scores of 4+, who are at extremely high risk of CNS disease and mortality, could be admitted for inpatient evaluation and treatment. Such stratification would enable a large proportion of CrAg-positive individuals to be easily managed as outpatients (43% [80/189] of CrAg-positive individuals from the screening studies included in our analysis had a CrAgSQ 1+ score) and intensive management to be focused on the smaller proportion of individuals at very high risk of complications to reduce the high mortality currently observed in this patient population.

Risk stratification based on CrAg quantification to guide differentiated care in patients presenting with clinical cryptococcal meningitis may also be possible. Extensive data show the strong association between higher baseline CrAg titers in both blood and CSF and subsequent mortality in patients undergoing treatment for HIV-associated cryptococcal meningitis. As new all-oral (4) and short-course (4, 21) treatment regimens for cryptococcal meningitis are developed, CrAg quantification could be used to define a population of patients who could be discharged from hospital early or be treated in ambulatory settings. Those with higher titers may be candidates for future adjuvant treatments (30) or longer courses of therapy.

Our study provides the first evidence for the diagnostic performance of the CrAgSQ in a CrAg screening program targeting individuals with CD4 cell counts of ≤200 cells/μl, and it also provides information to guide the interpretation of CrAgSQ scores in HIV-infected patients with cryptococcal infection. However, the utility of stratifying patient management based on these scores requires further evaluation in prospective trials. While we have shown associations between CrAgSQ scores and the key clinical variables of CNS disease and mortality, our analysis is limited by the relatively low levels of investigation for CNS disease at baseline and a lack of detailed information regarding adherence to treatment guidelines in the CrAg-positive outpatient population. Our analysis is also unable to provide any information regarding the potential impacts of introducing alternative management strategies according to CrAg-based risk stratification or the cost-effectiveness of these strategies. Although not yet confirmed, preliminary information from IMMY suggests the CrAgSQ test will cost in the range of $5 to $6. Finally, the CrAgSQ test identified some patients as positive at 1+ who had negative IMMY CrAg LFA and EIA results. While we have classified these as false positives, further CSF and clinical outcome data on this group are needed in order to determine whether such results represent early cryptococcal infection or not. Many of these false-positive samples had EIA optical density readings above zero but below the suggested cutoff, which may represent very low levels of cryptococcal antigenemia. A degree of disparity in test results is inevitable in samples with very low concentrations of the antigen at or near the limit of detection. Notably, none of the patients with positive CrAgSQ and negative IMMY CrAg LFA results developed cryptococcal disease, despite not receiving any antifungal therapy, suggesting that even if the results do represent very low CrAg titers, they are of limited clinical significance in individuals who initiate effective antiretroviral therapy.

In conclusion, the IMMY semiquantitative CrAgSQ cryptococcal antigen test had high sensitivity and specificity when compared to the current IMMY CrAg LFA and provided quantitative CrAg results that were associated with both CrAg titers derived from dilutional testing and clinical outcomes. The test provides an effective and practical method to stratify CrAg-positive patients according to CrAg levels and could provide the basis for differentiated management approaches to reduce the high mortality seen in HIV-positive patients with cryptococcal infection.

## Supplementary Material

Supplemental file 1
