# Supplementary material for: Evaluation of a Novel Semiquantitative Cryptococcal Antigen Lateral Flow Assay in Patients with Advanced HIV Disease
Source: J Clin Microbiol. 2020 Aug 24;58(9):e00441-20. doi: 10.1128/JCM.00441-20 (PMC7448662; doi:10.1128/JCM.00441-20)
Supplement: Supplemental file 1 [file JCM.00441-20-s0001.pdf]

Evaluation of a Novel Semi-quantitative Cryptococcal Antigen Lateral Flow Assay in Patients with Advanced HIV Disease. Joseph N Jarvis et al.

Supplementary Material

Supplementary table 1. Characteristics of samples with unreconciled discordant IMMY lateral flow assay (LFA) / enzyme immunoassay (EIA) and IMMY CrAgSQ test results

**1. CrAgSQ "False" positives (n=27)**			
	**CrAgSQ positive/LFA & EIA negative (n=27)**	**CrAgSQ positive/LFA positive (n=21)**	**P-value**
**CrAgSQ titer**	1+ (all cases)	1+ group for comparison	--
**Age**	42 years (IQR 39-47)	39 years (IQR 36-42)	0.1
**Sex**	67% (18) male	76% (16) male	0.5
**CD4 count**	60 cells/µL (IQR 46-146)	90 cells/µL (IQR 35-160)	0.9
**ART status**	19% (5) ART naive	19% (4) ART naive	0.7
**Prior CM**	0% (0) prior CM	9.5% (2) prior CM	0.1
**Baseline CM**	0% (0) baseline CM	4.8% (1) baseline CM	0.3
**Incident CM**	0% (0) incident CM	0% (0) incident CM	1.0
**Six-month outcome***	3.7% (1) dead<br>3.7% (1) lost to follow-up	9.2% (2) dead<br>0% (0) lost to follow-up	0.5
**2. CrAgSQ "False" negatives (n=1)**			
CrAgSQ negative, LFA positive in undiluted specimen only (titer reported as 1:2 due to required dilution with specimen reagent), EIA positive at optical density 0.393 (manufacturer's recommended cut-off 0.265). Forty-one year old female on ART, CD4 count 181 cells/µL. No history of previous cryptococcal disease, asymptomatic, declined baseline lumbar puncture, treated with oral fluconazole, alive at six-months follow-up, did not develop cryptococcal meningitis.			

* Patients were treated on the basis of their IMMY lateral flow result, therefore no CrAgSQ-positive IMMY lateral flow and enzyme immunoassay negative individuals received fluconazole therapy.

Supplementary Figure 1. Cryptococcal antigen screening and treatment algorithm

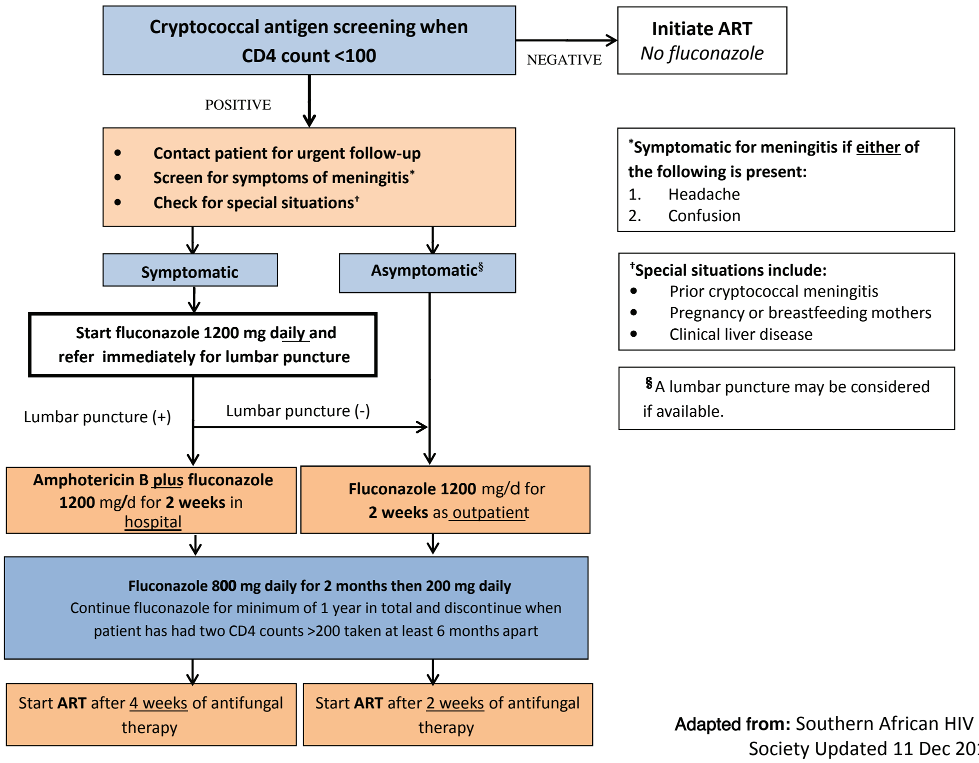

Adapted from: Southern African HIV Clinicians' Society Updated 11 Dec 2012

†Special situations

Prior cryptococcal meningitis
Patients with a history of cryptococcal meningitis *do not* need to be routinely screened. However, if the patient has been screened and has new symptoms of meningitis, he/ she will need to be evaluated for relapse disease and/or IRIS. If the patient does not have new symptoms, the health care provider should ensure that the patient has received or is receiving adequate maintenance therapy (fluconazole 200 mg until patient has had two CD4 counts >200 taken at least 6 months apart on ART).

Pregnancy or breastfeeding mothers
Because fluconazole can be harmful to a fetus, all women of childbearing age should have a pregnancy test. The risks, benefits and alternatives to fluconazole treatment should be discussed with the pregnant patient. Consultation with a medical practitioner experienced in the care of HIV-infected patients is recommended. Mothers who are breastfeeding also require consultation with an experienced medical practitioner as fluconazole can be transmitted through breast milk to the infant. Women of childbearing age who are not yet pregnant and are starting fluconazole treatment should be advised to avoid becoming pregnant during treatment.

Clinical liver disease
Patients with a history of liver disease or with evidence of clinical liver disease deserve careful monitoring because fluconazole may cause liver damage. Consultation with a physician experienced in the care of HIV-infected patients is recommended.

Reference: Guideline for the prevention, diagnosis and management of cryptococcal meningitis among HIV-infected persons: 2013 update. The Southern African HIV Clinicians Society. Southern African Journal of HIV Medicine; 14(2):a82. DOI: https://doi.org/10.4102/sajhivmed.v14i2.82. Licensed under CC Attribution 4.0

Photographs of IMMY CrAgSQ test strips

Photographs of CrAgSQ test strips will be provided by the corresponding author (Professor Joseph N Jarvis, email: joseph.jarvis@lshtm.ac.uk) on request.
